# Cause, effect, and remediation of melanin-associated bias in pulse oximetry

**DOI:** 10.1117/1.JBO.31.2.028002

**Published:** 2026-02-26

**Authors:** Kevin J. Benner, Neha N. Goel, Mark S. Rea

**Affiliations:** a Icahn School of Medicine at Mount Sinai, Light and Health Research Center, Department of Population Health Science and Policy, New York, New York, United States; b Icahn School of Medicine at Mount Sinai, Division of Pulmonary, Critical Care, and Sleep Medicine, Department of Medicine, New York, New York, United States

**Keywords:** oximetry, melanin bias, occult hypoxemia, precision

## Abstract

**Significance:**

Black patients are at greater risk than White patients for occult hypoxemia due to a melanin-associated bias in ${\mathrm{SpO}}_{2}$ readings from commercially available pulse oximeters that employ polychromatic light sources.

**Aim:**

We aim to demonstrate how melanin-associated inaccuracies in commercially available pulse oximeters increase the likelihood of occult hypoxemia for all patients and how monochromatic light sources could minimize melanin-associated occult hypoxemia.

**Approach:**

Published values of mean error (M $\left[{\mathrm{SpO}}_{2}-{\mathrm{SaO}}_{2}\right]$) and uncertainty (SD $\left[{\mathrm{SpO}}_{2}-{\mathrm{SaO}}_{2}\right]$) for Black and White patients were used to analytically model the risk of occult hypoxemia. Mean errors and uncertainties were also estimated for hypothetical patients with no skin melanin, which are directly comparable to values for pulse oximeters employing monochromatic light sources.

**Results:**

The analytically predicted risk of occult hypoxemia for Black patients relative to White patients was 2.1, consistent with published empirical findings. Oximeters unaffected by melanin in the skin would have smaller mean errors and uncertainties than current oximeters, significantly reducing the risk of occult hypoxemia in both Black patients (83% reduction) and White patients (65% reduction).

**Conclusions:**

Because everyone has melanin in their skin, the use of monochromatic light sources in pulse oximeters could significantly reduce the risk of occult hypoxemia for everyone.

## 1 Introduction

There has been heightened interest in the greater false negative (omission error) rates for diagnosing hypoxemia among individuals with dark skin using peripheral oxygen saturation measurements (${\mathrm{SpO}}_{2}$) from commercial pulse oximeters.^1^^–^^6^ A higher false negative rate means that these oximeters systematically overestimate blood oxygen levels in dark-skin patients. Empirically, this overestimation, or racial bias, results in as much as 2.5% higher ${\mathrm{SpO}}_{2}$ readings for self-reported Black patients than for self-reported White patients when their arterial blood oxygen saturation (${\mathrm{SaO}}_{2}$) readings are the same.^1^^,^^7^^,^^8^ Our previous analyses support the inference that the heightened false negative rate for dark-skin patients is a direct consequence of the polychromatic red and infrared (IR) light sources used in commercial pulse oximeters and the procedures required by the United States Food and Drug Administration (US FDA) for calibrating them.^9^

Two light sources are used in pulse oximetry, one red and one IR. The average changes in the red and IR light transmissions through (transmission mode) or from (reflectance mode) a peripheral part of the body (often a fingertip) are determined over several pulse cycles. The ratio (R) of these average changes is then used to estimate a patient’s blood oxygen saturation [Eq. (1)].

$$R\equiv \frac{\Delta T_{\text{red}}/{\overset{¯}{T}}_{\text{red}}}{\Delta T_{\mathrm{IR}}/{\overset{¯}{T}}_{\mathrm{IR}}},$$ (1)

where $\Delta T_{\text{red}}$ and $\Delta T_{\mathrm{IR}}$ are the changes in spectral transmissions of the red and the IR light sources over each pulse cycle, respectively. ${\overset{¯}{T}}_{\mathrm{IR}}$ and ${\overset{¯}{T}}_{\text{red}}$ are the average spectral transmissions over many pulse cycles.

For commercial oximeters, R values are converted into ${\mathrm{SpO}}_{2}$ values through coefficients empirically derived from an oximeter-specific calibration procedure [Eq. (2)] defined by the US FDA.

$${\mathrm{SpO}}_{2}\equiv C_{1}-C_{2}\cdot R≅{\mathrm{SaO}}_{2},$$ (2)

where $C_{1}$ and $C_{2}$ are empirically determined constants relating R to simultaneously collected ${\mathrm{SaO}}_{2}$ values.

The fundamentals of pulse oximetry are based on the Beer-Lambert law, which describes the attenuation of light through a substance.^10^^,^^11^ Light-emitting diodes (LEDs) are used in commercial pulse oximeters because they are inexpensive, rugged, produce little heat, and have a very long life. The spectral emissions of these LEDs are polychromatic with emitted optical power spread over a range of wavelengths (typically >20  nm). From the Beer-Lambert law, when polychromatic spectra are transmitted through melanin in the epidermis, there is a spectral shift of the radiant energy to longer wavelengths.^9^ The spectral absorption profile of melanin progressively decreases as wavelength increases, so the polychromatic LED spectra will have greater transmission through melanin at longer wavelengths, resulting in a spectral shift in the light exiting the epidermal layer. Importantly, the greater the melanin concentration, the greater the spectral shift.

According to the Beer-Lambert law, because spectral transmittance changes exponentially with pigment density, the ratio of ratios used to calculate R, and thus ${\mathrm{SpO}}_{2}$, does not factor out the different, melanin-associated changes in the spectral transmissions of the constituent wavelengths of two polychromatic sources. If the absorption spectrum of melanin were flat (i.e., absorbed equally at all wavelengths of the emission), the influence of melanin would be corrected by the ratio of ratios. The spectral shift for the polychromatic red light is particularly important. When the spectrum of the red light shifts as it passes through melanin, the difference in red-light absorption by oxygenated and deoxygenated blood is reduced, and the R ratio [Eq. (1)] is reduced. Consequently, the smaller the R value, the greater the ${\mathrm{SpO}}_{2}$ value [Eq. (2)] and the greater the false-negative error rate (i.e., overestimation of blood oxygen saturation). If monochromatic light sources were used in pulse oximeters, it would be impossible for the spectral power distributions of the oximeter’s two light sources to shift, thereby reducing, if not eliminating, the melanin-associated bias inherent with pulse oximeters that use polychromatic light sources.

The US FDA calibration procedure is usually based upon a preponderance (≈85%) of light-skin subjects. The melanin-associated spectral shift to longer wavelengths for polychromatic light sources means that patients with skin darker than that of the usual calibration sample will, for the same ${\mathrm{SaO}}_{2}$, provide systematically higher ${\mathrm{SpO}}_{2}$ values. This is, of course, now widely observed. However, changes to the distribution of skin types will not eliminate melanin-associated biases; it would only change the distribution of skin types that will have inaccurate ${\mathrm{SpO}}_{2}$ measurements.

Although not widely recognized, melanin-associated bias is bidirectional. Instead of a higher false-negative rate for dark-skin patients when light-skin subjects are used for the pulse oximeter calibration, a calibration based largely upon a sample of dark-skin subjects would necessarily increase the false-positive (commission error) rate for those with light skin. In short, melanin-associated bias can never be avoided when polychromatic light sources are used in pulse oximeters, no matter what cohort is used in the calibration procedure.

Melanin is not uniformly distributed in the epidermis but is pseudo-randomly distributed as discrete, spectrally absorbing melanin molecules called epidermal melanin units (EMUs).^12^ Moreover, the epidermal layer is not planar, having invaginations and protrusions across its boundary layers. The distribution of EMUs and the spatial features of the epidermal layer naturally result in different optical path lengths through the spectrally absorbing melanin as the entrance angle of the light through the skin changes. Slight movements of the pulse oximeter LED probe at a peripheral measurement site would consequently lead to slightly different values of R, thus increasing ${\mathrm{SpO}}_{2}$ measurement variance for the same overall density of EMUs in the epidermis.

In addition to melanin-associated bias, higher concentrations of melanin in the skin also lead to greater measurement variance because there is a greater number of EMUs in the epidermis. Studies have shown that ${\mathrm{SpO}}_{2}$ measurement variance from self-reported Black patients is up to 5% higher than that for self-reported White patients.^1^^,^^7^^,^^8^ Consistent with these observations, we experimentally showed that the variance for three independent measurements of each subject’s R [Eq. (1)] systematically increased according to skin pigment density.^9^^,^^13^

### 1.1 Monochromatic Light Sources

If monochromatic light sources were used in oximeters, both melanin-associated bias and melanin-associated variance would be reduced. Because there is no radiant energy at any other wavelength, the spectral shift due to melanin is simply eliminated. Further, the heightened variance in the ${\mathrm{SpO}}_{2}$ measurements for dark-skin patients would also be reduced because the additional spectral energy in a polychromatic source could not contribute to the measurement variance. Only the attenuated intensities of the monochromatic red and IR lights could affect bias and variance.

Importantly, if monochromatic light sources were used in pulse oximetry, the incidence of missed diagnoses of hypoxemia would also decrease if a clinical diagnosis were dependent only upon ${\mathrm{SpO}}_{2}$ measurements, because the ${\mathrm{SpO}}_{2}$ measurements would be more accurate than measurements using current, polychromatic oximeters. Occult hypoxemia occurs when ${\mathrm{SaO}}_{2}$ falls below 88% but ${\mathrm{SpO}}_{2}$ indicates normal levels (≥92%). An ${\mathrm{SaO}}_{2}$ value of 88% is clinically important because it represents the beginning of the steep portion of the oxygen–hemoglobin dissociation curve, where small changes in oxygen partial pressure result in significant changes in oxygen saturation and, thus, oxygen delivery to tissues. Since most commercial pulse oximeters using polychromatic LEDs provide systematically biased and less certain ${\mathrm{SpO}}_{2}$ readings for patients with relatively high melanin concentration in their skin, the likelihood of occult hypoxemia is necessarily greater for these individuals. As we subsequently show here, the relatively small differences in bias and variance between dark skin and light skin have led to a relatively large difference in the incidence of occult hypoxemia for these two populations. It is also worth pointing out that if monochromatic light sources were used in pulse oximeters, the pressure on the US FDA to change their current calibration procedures in consideration of patient skin pigmentation would be relieved.

### 1.2 Study Goals

The dual goals of the present study were, first, to demonstrate how melanin-associated inaccuracies generated by commercially available pulse oximeters increase the likelihood of occult hypoxemia for both Black and White patients and, second, to examine how monochromatic light sources would minimize melanin-associated occult hypoxemia. To achieve these goals, we first modeled occult hypoxemia probabilities based on bias and precision measurements of commercial oximeters reported in the literature. We then examined the literature to estimate melanin concentration in the epidermis of dark- and light-skin individuals so that we could determine how both bias and precision were functionally related to melanin concentration.

## 2 Materials and Methods

### 2.1 Definition of Mean Error and Uncertainty

The accuracy of pulse oximetry measurements is usually characterized both in terms of (1) “bias,” which is the mean (M) difference between arterial and peripheral blood oxygen saturation measurements $\left[M({\mathrm{SpO}}_{2}-{\mathrm{SaO}}_{2})\right]$, and (2) “precision,” which is the standard deviation (SD) of “bias” [SD $\left({\mathrm{SpO}}_{2}-{\mathrm{SaO}}_{2}\right)$]. These two descriptive statistics of accuracy are commonly combined into a single metric, accuracy root mean square (ARMS), where $\mathrm{ARMS}=\surd [M({\mathrm{SpO}}_{2}-{\mathrm{SaO}}_{2}{)}^{2}+\mathrm{SD}({\mathrm{SpO}}_{2}-{\mathrm{SaO}}_{2}{)}^{2}]$. To avoid possible confusion with racial bias, the term mean error will be used here instead of “bias.” Because “low precision” is desirable in the definition of accuracy (contrary to the colloquial usage of “high precision”), the term uncertainty is used here in reference to the standard deviation of error.

### 2.2 Mean Error and Uncertainty Estimates for Black and White Patients

Very recently, Parr and colleagues^3^ performed a comprehensive review and meta-analysis to estimate the likelihood of occult hypoxemia as a function of race. In their study, they also reported pooled estimates of mean error and uncertainty for Black and White patients. Their reported pooled values, based upon the most recent published studies (2021–2023), showed a mean error ($M[{\mathrm{SpO}}_{2}-{\mathrm{SaO}}_{2}]$) of 1.16 for Black patients and 0.44 for White patients, and uncertainty values (SD $\left[{\mathrm{SpO}}_{2}-{\mathrm{SaO}}_{2}\right]$) of 4.48 for Black patients and 3.80 for White patients.^3^

### 2.3 Estimates of Occult Hypoxemia from Mean Error and Uncertainty Values

Bangash and colleagues^14^ reported that the difference between ${\mathrm{SpO}}_{2}$ and ${\mathrm{SaO}}_{2}$ is normally distributed. Under this assumption and using the mean error and uncertainty values reported by Parr et al.,^3^ we used Eq. (3) to calculate the probability that White and Black patients with a given ${\mathrm{SpO}}_{2}$ value would be expected to have occult hypoxemia.

$$p_{\mathrm{OH}}=1-\Phi (z)$$

where

$$z=\frac{{\mathrm{SpO}}_{2}-{\mathrm{SaO}}_{2}-\mu }{\sigma }$$ (3)

For a given ${\mathrm{SpO}}_{2}$, $p_{\mathrm{OH}}$ (probability of occult hypoxemia) is the proportion of a population (e.g., self-reported Black or self-reported White patients) with occult hypoxemia, ${\mathrm{SaO}}_{2}$ is the occult hypoxemia cutoff value of 88%, Φ(z) is the cumulative distribution function for the standard normal distribution, and μ and σ are the mean error and uncertainty, respectively.

The risk of occult hypoxemia for Black patients, relative to White patients, at a given ${\mathrm{SpO}}_{2}$ is determined by dividing $p_{\mathrm{OH}}$ for Black patients by $p_{\mathrm{OH}}$ for White patients. The absolute risk of occult hypoxemia is determined by multiplying the $p_{\mathrm{OH}}$ distributions for each patient category by the probability distribution of ${\mathrm{SpO}}_{2}$ and integrating over the range of 92% to 100% ${\mathrm{SpO}}_{2}$.

### 2.4 Mean Error and Uncertainty as a Function of Melanin Content

Since mean error and uncertainty are positively correlated with melanin content,^9^^,^^13^ ${\mathrm{SpO}}_{2}$ measurement inaccuracy would necessarily be less for individuals with no melanin in the skin; however, no pulse oximeter measurements from individuals without melanin were found in the literature. Acknowledging the necessary cautions associated with extrapolations, it is logically possible to estimate mean error and uncertainty values for individuals without melanin from the observed mean error and uncertainty values for White and Black patients reported by Parr et al.^3^ if the melanin content in the skin of White and Black patients is known. Alaluf et al.^15^ reported objective measurements of melanin content for Africans and Europeans. Under the assumption that the reported melanin concentration values for those two groups correspond with the levels of skin pigmentation for the Black and White patients reported by Parr et al.,^3^ it is possible to functionally relate both mean error and uncertainty to melanin concentration, and then, by linear extrapolation, estimate mean error and uncertainty for individuals with no melanin in the skin. Since measurements of R from monochromatic oximeters would be unaffected by melanin, the extrapolated values of mean error and uncertainty for individuals without melanin in the skin would be the same as those for monochromatic oximeters. The data used in the modeling methods described in Secs. 2.1–2.4 are summarized in Table 1.

**Table 1:** Summary of data from literature used in the modeling.

Data used in analysis	Source	Value
Mean error of pulse oximetry for White patients	Parr et al.^3^	0.44%
Mean error of pulse oximetry for Black patients	Parr et al.^3^	1.16%
Standard deviation of error of pulse oximetry for White patients	Parr et al.^3^	3.80%
Standard deviation of error of pulse oximetry for Black patients	Parr et al.^3^	4.48%
Risk of occult hypoxemia for Black patients, relative to White patients	Parr et al.^3^	1.84
Probability distribution of ${\mathrm{SpO}}_{2}$ for all patients	Bangash et al.^14^	See Fig. 2(b)
Melanin density for White subjects	Alaluf et al.^15^	19.9 μg/mg
Melanin density for Black subjects	Alaluf et al.^15^	40.1 μg/mg

## 3 Results

### 3.1 Likelihood of Occult Hypoxemia

The mean error and uncertainty values reported by Parr et al.^3^ and Eq. (3) were used to calculate the proportions of Black and White patients with occult hypoxemia. For example, Eqs. (4) and (5) [both calculated from Eq. (3)] show the calculation for ${\mathrm{SpO}}_{2}=94\%$:

$$p_{\mathrm{OH}\_\text{Black}}=1-\Phi (\frac{94-88-1.16}{4.48})=0.14$$ (4)

$$p_{\mathrm{OH}\_\text{White}}=1-\Phi (\frac{94-88-0.44}{3.80})=0.07$$ (5)

**Fig. 1:**
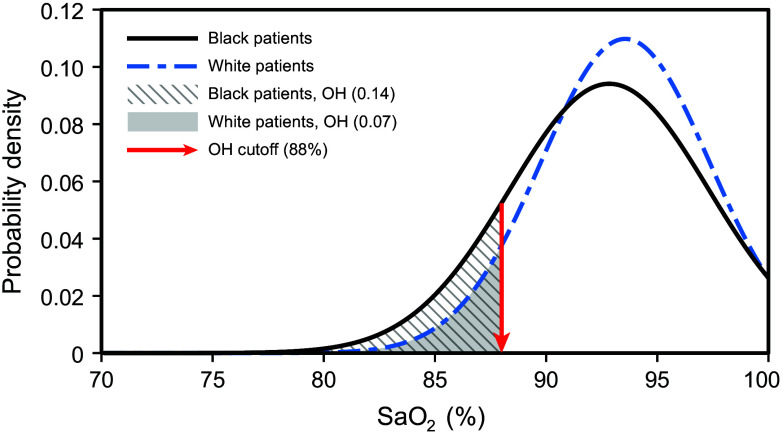
Proportions of Black and White patients predicted to exhibit occult hypoxemia (${\mathrm{SaO}}_{2}<88\%$) at the exemplary ${\mathrm{SpO}}_{2}$ value of 94%. The predicted Gaussian distributions of ${\mathrm{SaO}}_{2}$ measurements for Black and White patients are based on the mean error and uncertainty values for the two groups reported by Parr et al.^3^ The proportions of White and Black patients predicted to exhibit ${\mathrm{SaO}}_{2}<88\%$ when ${\mathrm{SpO}}_{2}=94\%$ (occult hypoxemia) are illustrated by the solid gray and hatched gray areas, respectively.

In this example, the relative risk of being misdiagnosed would then be 2.0 (0.14/0.07) for Black patients compared to White patients if, for both populations, ${\mathrm{SpO}}_{2}=94\%$ (Fig. 1).

**Fig. 2:**
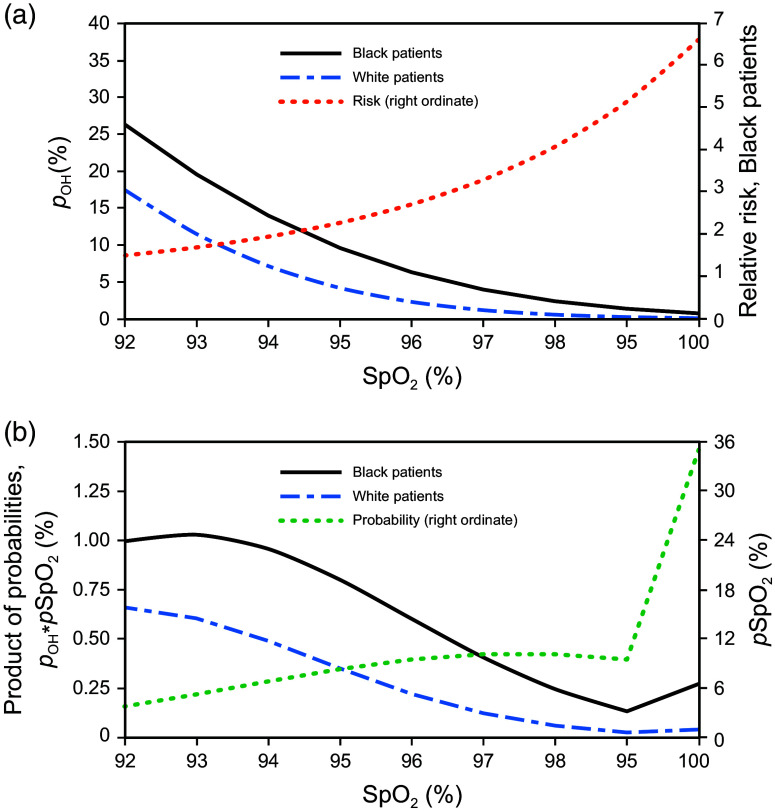
(a) Probability of occult hypoxemia ($p_{\mathrm{OH}}$; left ordinate) for Black (solid line) and White (dash-dotted line) patients as a function of ${\mathrm{SpO}}_{2}$, along with the relative risk (right ordinate) for Black patients (dotted line). (b) Probability distribution of measured ${\mathrm{SpO}}_{2}$ values from all patients ($p_{\mathrm{SpO}2}$; dotted line, right ordinate), adapted from Bangash et al.^14^ The probabilities of occult hypoxemia (left ordinate) for Black (solid line) and White (dash-dotted line) patients are obtained from the product of $p_{\mathrm{SpO}2}$ and the $p_{\mathrm{OH}}$ functions shown in Fig. 2(a).

Values of $p_{\mathrm{OH}}$ (left ordinate) for ${\mathrm{SpO}}_{2}$ values between 92% and 100% were calculated for Black and White patients and are illustrated in Fig. 2(a). From these functions, the risk of occult hypoxemia for Black patients relative to White patients ($p_{\mathrm{OH}\_\text{Black}}/p_{\mathrm{OH}\_\text{White}}$) as a function of ${\mathrm{SpO}}_{2}$ is also illustrated in Fig. 2(a) (right ordinate); the relative risk changes from about 1.5 at 92% ${\mathrm{SpO}}_{2}$ to about 6.6 at 100% ${\mathrm{SpO}}_{2}$. A distribution of the probability of a patient having a given ${\mathrm{SpO}}_{2}$ ($p_{\mathrm{SpO}2}$) is adapted from data presented by Bangash et al.^14^ and shown in Fig. 2(b). This distribution represents a normal distribution with a mean of 97.5% and a standard deviation of 3.9%. Because pulse oximeters do not report ${\mathrm{SpO}}_{2}$ over 100%, all values over 100% ${\mathrm{SpO}}_{2}$ are assigned to a value of 100%. This results in a significant change in slope from 99% to 100% saturation, as shown in Fig. 2(b); this discontinuity is also shown in the original data reported by Bangash et al.

The areas under each of the two $p_{\mathrm{OH}}$ curves in Fig. 2(b) represent the absolute risk of occult hypoxemia by patient category, which is 0.054 for Black patients and 0.026 for White patients. The ratio of the areas under these curves represents the relative risk for Black patients, which is 2.1.

### 3.2 Melanin-free Estimates of Mean Error and Uncertainty

**Fig. 3:**
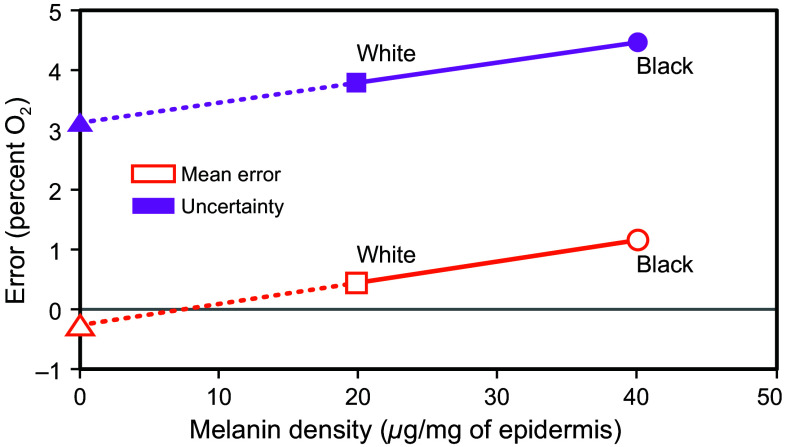
Estimates of melanin-free mean error and uncertainty values to demonstrate the theoretical performance capabilities of a narrowband oximeter, extrapolated from the average values reported by Parr et al.^3^ and the physical measurements of skin melanin density reported by Alaluf et al.^15^

Based upon the mean error and uncertainty values for Black and White patients reported by Parr et al.,^3^ together with the reported melanin content for European and African subjects from Alaluf et al.,^15^ Fig. 3 shows the estimated mean error and uncertainty values for individuals without melanin in the skin and for pulse oximeters using monochromatic light sources. Based upon this approach, the melanin-free mean error value would be −0.27% (roughly 0%), and the melanin-free uncertainty value would be 3.1%.

Using these values for mean error (−0.27%) and uncertainty (3.1%), the predicted occult hypoxemia rate would be 0.086 for both Black and White patients at a ${\mathrm{SpO}}_{2}$ of 92%, and an absolute risk of just 0.009 for both groups. If monochromatic light sources were used in oximeter probes, occult hypoxemia prevalence would be reduced by 83% for Black patients (from 0.054 to 0.009) and by 65% for White patients (from 0.026 to 0.009).

## 4 Discussion

A primary focus of our paper was demonstrating why melanin-associated inaccuracies, both mean error and uncertainty, generated by commercially available pulse oximeters using polychromatic LED light sources, increase the likelihood of occult hypoxemia for all patients and by a greater amount in Black patients.

Using the pooled estimates of mean error and uncertainty from Parr et al.,^3^ along with the ${\mathrm{SpO}}_{2}$ distribution provided by Bangash et. al.,^14^ our analytical procedures revealed a relative risk of 2.1 for occult hypoxemia in Black patients using commercial pulse oximeters. The results of this analytical modeling approach agree closely with the Parr et al. empirical estimate using pooled values of occult hypoxemia from the literature, which showed the relative risk of occult hypoxemia for Black patients relative to White patients to be 1.84. Since our model agrees with the empirical results, it can be used to estimate how occult hypoxemia rates would differ from current empirical estimates for oximeters with different values of mean error and uncertainty.

Other studies are in general agreement with those two independent estimates stemming from Parr. Henry et al.^16^ reported that the absolute risk to White and to Black patients for experiencing occult hypoxemia during hospitalization was 0.036 and 0.062, respectively, if only one measurement is obtained (a 1.72 relative risk for Black patients). They also reported that rates of occult hypoxemia for White and Black patients were 0.0122 and 0.0182, respectively, when multiple ${\mathrm{SpO}}_{2}$ measurements were taken (a 1.49 relative risk for Black patients). Chesley et al.^17^ reported slightly greater absolute risks of occult hypoxemia for White patients (0.029) and for Black patients (0.079), resulting in a relative risk of 2.7 for Black patients. All of these values are consistent with earlier findings by Sjoding et al.^1^ Still other studies report the relative risk of occult hypoxemia for Black patients to range from 1.7 to over 2.0.^5^^,^^18^

Our previous theoretical and empirical results demonstrate that the combined effect of polychromatic light sources in pulse oximeters and the procedures used by the US FDA to calibrate commercial oximeters creates melanin-associated inaccuracies. The analyses presented here reinforce our earlier findings. By using monochromatic light sources, melanin-associated inaccuracies (mean error and uncertainty) governed by the Beer-Lambert law would be eliminated. Thus, only small effects that may be melanin-associated, such as scattering or signal-to-noise ratio differences, would remain, so overall melanin-associated inaccuracies would be significantly reduced. More significantly, if monochromatic light sources were used in pulse oximeters, the incidence of occult hypoxemia would be lower for everyone.

Technical barriers to the widespread adoption of monochromatic sources may exist. Laser diodes are relatively inexpensive and operate similarly to LEDs that are commonly used today in pulse oximeters, but the use of lasers would require consideration of the beam size and irradiance at the patients’ skin to ensure thermal safety. Interference filters may be used to narrow the emission spectrum of LEDs, but they are expensive, may not produce a truly monochromatic spectrum, and their performance is dependent on the angle of the incident light. In addition, oximeter manufacturers must be motivated to develop these improved oximeters, which may require changes to regulations requiring lower error or decreased racial bias.

### 4.1 Conclusion

The melanin-associated inaccuracies of ${\mathrm{SpO}}_{2}$ measurements from practically all commercial pulse oximeters are of two types. The difference in mean error ($M[{\mathrm{SpO}}_{2}-{\mathrm{SaO}}_{2}]$) between Black and White patients in ${\mathrm{SpO}}_{2}$ measurements is small (<2.5%) and is now well recognized.^19^ The difference in uncertainty ($\mathrm{SD}[{\mathrm{SpO}}_{2}-{\mathrm{SaO}}_{2}]$) of ${\mathrm{SpO}}_{2}$ measurements is also small (<5%), but it is not as widely recognized.^13^ Most importantly, however, these two small sources of inaccuracy contribute to a greater relative risk of occult hypoxemia for Black patients compared to White patients (relative risk = 2.1) when using commercial oximeters calibrated according to the US FDA procedures.

Beyond the greater risk of occult hypoxemia for Black patients than for White patients, our analyses show that melanin, found in everyone’s skin, creates systematic inaccuracies for every patient when using commercially available pulse oximeters that employ polychromatic light sources. If monochromatic spectra were employed, the melanin-associated ${\mathrm{SpO}}_{2}$ measurement inaccuracies would be significantly reduced, and the likelihood of occult hypoxemia would also be reduced for every patient. The effects of melanin, and the benefit provided by monochromatic emitters for eliminating those effects, should be further studied in other medical optical measurement applications currently employing polychromatic emitters, such as near-IR spectroscopy.^20^

## Data Availability

The data underlying the results presented in this paper are available to subscribers from the cited sources and may be publicly available from the National Library of Medicine. The code underlying the results presented in this paper is not publicly available at this time but may be obtained from the authors upon reasonable request.
